# Third-Line and Later Susceptibility-Guided *Helicobacter pylori* Eradication Therapies: A Multicenter Study of Vonoprazan–Amoxicillin–Sitafloxacin/Rifabutin Regimens

**DOI:** 10.3390/jcm15020434

**Published:** 2026-01-06

**Authors:** Hideki Mori, Yoshimasa Saito, Hiroko Ando, Tatsuhiro Masaoka, Juntaro Matsuzaki, Masaru Nakano, Takanori Kanai

**Affiliations:** 1 Division of Gastroenterology and Hepatology, Department of Internal Medicine, Keio University School of Medicine, Tokyo 160-8582, Japan; ysaito@keio.jp (Y.S.);; 2 Division of Pharmacotherapeutics, Keio University Faculty of Pharmacy, Tokyo 105-8512, Japan; 3 Department of Gastroenterology, Kitasato University Kitasato Institute Hospital, Tokyo 108-8642, Japan; msnakano@insti.kitasato-u.ac.jp; 4 Center for Endoscopy, Kawasaki Municipal Hospital, Kawasaki 210-0013, Japan

**Keywords:** vonoprazan, amoxicillin, sitafloxacin, rifabutin, susceptibility-guided therapy

## Abstract

**Background/Objectives**: Although vonoprazan-based triple therapy has improved the first- and second-line *Helicobacter pylori* eradication rates, a subset of patients still require third-line or later treatments. The present study aimed to evaluate the efficacy and safety of susceptibility-guided eradication strategies from third-line or later treatments in a multicenter setting. **Methods**: This retrospective multicenter study (2019–2024) enrolled 94 patients who had failed second-line eradication therapy and underwent *H. pylori* isolation and susceptibility testing. Based on sitafloxacin sensitivity, patients received vonoprazan, amoxicillin, and sitafloxacin (VAS) if sensitive, or vonoprazan, amoxicillin, and rifabutin (VAR) if resistant. Altogether, 75 patients received treatment according to this protocol. **Results**: Among the 75 patients, 61.3% were sitafloxacin-sensitive (VAS group), and 38.7% were resistant (VAR group). All strains were rifabutin-sensitive. The overall eradication rates were 92.0% and 95.8% in the intention-to-treat and per-protocol analyses, respectively. Adverse events occurred in 17.3% of cases. One patient in the VAR group discontinued therapy due to dizziness, whereas all other adverse events were mild and did not require treatment cessation. Subgroup analysis showed eradication rates of 93.5% (43/46) and 89.7% (26/29) for the VAS and VAR groups, respectively. The eradication rate for third-line therapy was 96.2% (50/52), whereas that for fourth-line therapy was 85.7% (18/21). Fifth-line therapy showed an eradication rate of 50.0% (1/2). **Conclusions**: Susceptibility-guided vonoprazan-based regimens from the third-line treatment onward achieved high eradication and tolerability in a multicenter cohort. This approach may offer a valuable treatment option for patients with refractory *H. pylori* infections.

## 1. Introduction

*Helicobacter pylori* infection is a chronic disease of the stomach. *H. pylori* eradication prevents recurrence of peptic ulcers and reduces the incidence of gastric cancer. It also alleviates dyspeptic symptoms in a subset of patients with functional dyspepsia. Therefore, eradication therapy is strongly recommended [1,2,3,4].

In Japan, the first-line eradication regimen covered by the national insurance comprises a proton pump inhibitor (PPI) or vonoprazan in combination with amoxicillin and clarithromycin, whereas the second-line regimen consists of PPI or vonoprazan plus amoxicillin and metronidazole [5,6,7]. However, 1–3% of patients fail to achieve eradication even after undergoing the second-line treatment. Third-line or subsequent eradication therapies are not covered by insurance, and no optimal regimen has been established to date. Likewise, in regions outside Japan, overlapping resistance to multiple antibiotics is frequently observed in patients receiving third-line or later eradication therapies, highlighting the ongoing need to establish effective treatment regimens across different geographic settings [8,9,10,11].

In *H. pylori* eradication, acid-suppressive agents are administered to stabilize the serum concentration of antibiotics [12]. Compared with conventional proton pump inhibitors (PPIs), vonoprazan provides more rapid, potent, and sustained acid suppression, with less interindividual variability related to CYP2C19 polymorphisms [13,14,15]. These pharmacological advantages are particularly relevant in rescue settings, where maintaining a stable intragastric pH is critical for maximizing the efficacy of antibiotics against resistant *H. pylori* strains. Vonoprazan, a novel acid suppressant that inhibits the H^+^/K^+^-ATPase via a potassium-competitive mechanism, was launched in Japan in 2015 and has since contributed to improved eradication outcomes when incorporated into *H. pylori* eradication regimens [16,17].

In particular, increasing resistance to clarithromycin and metronidazole has been directly associated with insufficient eradication rates, underscoring the need to develop eradication regimens centered on antibiotics that do not include these agents [18,19,20]. Consequently, fluoroquinolones and rifabutin have been adopted as key alternative antibiotics for rescue therapy [21,22,23,24,25,26,27,28,29].

In Japan, regimens containing sitafloxacin, a fluoroquinolone available for clinical use, have been actively developed following evidence from a multicenter prospective randomized controlled trial demonstrating their superior efficacy compared with levofloxacin-based regimens, which belong to the same antibiotic class [30]. More recently, the triple regimen of vonoprazan, amoxicillin, and sitafloxacin (VAS regimen) has been shown to be more effective than conventional proton pump inhibitor-based therapies [31,32]. In particular, the VAS regimen reportedly achieves reliable eradication when the *H. pylori* strain is sensitive to sitafloxacin [31].

In contrast, rifabutin-containing regimens have been employed in Japan mainly as salvage therapy for patients with failure of sitafloxacin-based regimens; however, their use has remained relatively limited in routine clinical practice [33,34]. Rifabutin resistance is rare [24,35], and the triple regimen of vonoprazan, amoxicillin, and rifabutin (VAR regimen) has achieved eradication rates exceeding 90% as a fourth-line therapy [34].

Although susceptibility-guided therapy is theoretically advantageous and recommended by international guidelines [6,10,18,36,37], its application in routine clinical practice remains limited, particularly beyond second-line eradication therapy. Moreover, empirical rescue therapy continues to be widely used in real-world practice, despite the growing concern that repeated exposure to ineffective antibiotics may further promote antimicrobial resistance and compromise subsequent treatment options [27,38,39,40]. These gaps in evidence highlight the need for robust real-world data evaluating the effectiveness and safety of susceptibility-guided eradication strategies in patients with refractory *H. pylori* infection [41,42].

In this context, evaluating a practical susceptibility-guided algorithm that can be readily implemented in routine clinical settings is of particular clinical relevance. Stratifying patients based on sitafloxacin susceptibility allows for rational selection between sitafloxacin- and rifabutin-containing regimens, thereby optimizing antibiotic use while minimizing unnecessary exposure to ineffective agents. However, multicenter data assessing the real-world performance of such individualized strategies in third-line and subsequent eradication therapy remain limited [8]. Therefore, the present multicenter retrospective study aimed to assess the efficacy and safety of susceptibility-guided vonoprazan-based eradication therapy from the third-line setting onward and to clarify its clinical utility in routine practice.

## 2. Materials and Methods

### 2.1. Study Design

This multicenter, retrospective observational study was conducted at Keio University Hospital and Kitasato University Kitasato Institute Hospital. The present study aimed to evaluate the eradication rate of individualized *H. pylori* eradication therapy based on antimicrobial susceptibility test results and to identify factors associated with eradication success.

### 2.2. Study Population

#### 2.2.1. Inclusion Criteria

Eligible patients met all of the following criteria: (1) underwent third-line or subsequent *H. pylori* eradication therapy between November 2019 and December 2024 at Keio University Hospital or Kitasato University Kitasato Institute Hospital; (2) underwent antimicrobial susceptibility testing prior to third-line eradication therapy; (3) received individualized eradication therapy according to the study protocol, in which the treatment regimen was selected based on the sitafloxacin minimum inhibitory concentration (MIC) results (MIC ≥ 0.12 µg/mL: VAR regimen; MIC < 0.12 µg/mL: VAS regimen); and (4) age of ≥18 years.

#### 2.2.2. Exclusion Criteria

Patients were excluded if they met any of the following criteria: (1) known allergy to sitafloxacin or rifabutin; (2) had not received therapy according to the abovementioned individualized protocol; (3) refused the use of their clinical data; (4) had missing or unassessable eradication outcomes; and (5) had incomplete information regarding the eradication regimen or antibiotic dosage.

### 2.3. Antimicrobial Susceptibility Testing

*H. pylori* strains were isolated from gastric biopsy specimens obtained during upper gastrointestinal endoscopy before initiation of third-line or subsequent eradication therapy. Antimicrobial susceptibility testing was performed using the agar dilution method, which is considered a reference standard for determining minimum inhibitory concentrations (MICs) of *H. pylori*. All susceptibility testing was outsourced to a single commercial laboratory (LSI Medience Corporation, Tokyo, Japan), where a standardized and unified protocol was applied throughout the study period. Susceptibility results were reported as MIC values for each antimicrobial agent. The MIC cutoff values used to define resistance were 0.12, 0.25, 0.06, 1, and 8 µg/mL for sitafloxacin, rifabutin, amoxicillin, clarithromycin, and metronidazole, respectively [38]. All participating institutions adhered to identical criteria for interpretation of susceptibility results, ensuring consistency across centers.

### 2.4. Treatment Regimens

Treatment selection was based on the results of sitafloxacin susceptibility testing and followed a predefined susceptibility-guided algorithm applied uniformly across all participating institutions. Patients infected with *H. pylori* strains showing sitafloxacin minimum inhibitory concentration (MIC) values of <0.12 µg/mL were assigned to the VAS regimen (vonoprazan, amoxicillin, and sitafloxacin), whereas those with MIC values of ≥0.12 µg/mL were assigned to the VAR regimen (vonoprazan, amoxicillin, and rifabutin). This MIC-based criterion was predefined and intended to be applied uniformly, including in cases with MIC values near the cutoff. The protocol-based treatment recommendation was explained to all patients, and although both regimens were presented, treatment was generally selected in accordance with the predefined algorithm. In a limited number of cases, the final treatment choice differed from the protocol-based recommendation after discussion with the patient. These cases were defined as protocol deviations and were excluded from the present analysis.

For the VAR regimen, patients received vonoprazan 20 mg twice daily, amoxicillin 500 mg four times daily, and rifabutin 150 mg once daily for 7 days [34]. For the VAS regimen, patients received vonoprazan 20 mg twice daily, amoxicillin 500 mg four times daily, and sitafloxacin 100 mg twice daily for 7 days [31]. In principle, no deviations from the predefined treatment algorithm were permitted; patients who did not receive therapy consistent with this protocol were excluded from the present analysis, as described in the exclusion criteria. Patients were instructed both verbally and in writing to adhere to the dosing schedule, and treatment adherence was assessed by patient interview at follow-up visits.

### 2.5. Confirmation of Eradication

Eradication was confirmed 12 weeks after completion of therapy using either the ^13^C-urea breath test or *H. pylori* stool antigen test.

The ^13^C-urea breath test was performed using ^13^C-labeled urea (UBIT; Otsuka Pharmaceutical Co., Ltd., Tokyo, Japan). After an overnight fast, patients ingested 100 mg of ^13^C-urea dissolved in water. Breath samples were collected at baseline and 20 min after ingestion, according to a standardized protocol. The ^13^CO_2_ concentration in breath samples was measured using an infrared spectrometer (UbiT-IR 200; Otsuka Pharmaceutical Co., Ltd., Tokushima, Japan). The delta over baseline (Δ^13^CO_2_) value was calculated, and a cutoff value of ≥2.5‰ was considered positive for *H. pylori* infection, in accordance with established validation studies [6,18,43,44,45].

The stool antigen test was conducted using a monoclonal antibody-based enzyme immunoassay, Testmate Pylori Antigen EIA (Wakamoto Pharmaceutical Co., Ltd., Tokyo, Japan). Stool antigen positivity was defined according to the manufacturer’s recommended criteria, with an optical density value ≥ 0.1 considered positive, as described in a previous validation study [46,47].

### 2.6. Endpoints

#### 2.6.1. Primary Endpoint

The primary endpoint was the *H. pylori* eradication rate achieved with individualized therapy based on sitafloxacin susceptibility.

#### 2.6.2. Secondary Endpoints

The secondary endpoints were as follows: (i) identification of factors associated with successful eradication [antibiotic resistance, regimen type (VAS vs. VAR)]; (ii) incidence of adverse events; and (iii) number of prior eradication attempts.

### 2.7. Data Collection

Clinical data were extracted from the electronic medical records at each participating institution using a standardized case report form. These data included patient demographics, comorbidities, antimicrobial susceptibility results, eradication regimen details (drug names, doses, frequencies, and duration), number of prior eradication attempts, and adverse events.

Adverse events were assessed retrospectively based on a review of electronic medical records and direct patient interviews conducted at follow-up visits. No standardized adverse event questionnaire was used. Adverse events were recorded when reported spontaneously by patients or when noted by physicians during routine clinical practice.

All data were anonymized and assigned unique study identifiers prior to the analysis.

### 2.8. Statistical Analysis

Statistical analyses were performed using SPSS Statistics version 29 (IBM Corp., Armonk, NY, USA). Continuous variables were expressed as mean ± standard deviation and compared using Student’s *t*-test. Categorical variables were expressed as counts and percentages and analyzed using Fisher’s exact test. Eradication rates were calculated in the intention-to-treat (ITT) and per-protocol (PP) populations with two-sided 95% confidence intervals (CIs) using the exact (Clopper–Pearson) method. The ITT population included all patients who received at least one dose of the eradication therapy according to the study protocol. The PP population included patients who completed the assigned treatment regimen and underwent post-treatment eradication assessment, excluding those who discontinued therapy prematurely or were lost to follow-up. A two-sided *p*-value of <0.05 was considered statistically significant. Because of the limited sample size and the small number of eradication failure events, multivariate analyses were not performed to avoid model overfitting and unstable estimates.

## 3. Results

### 3.1. Study Flow and Population

**Figure 1:**
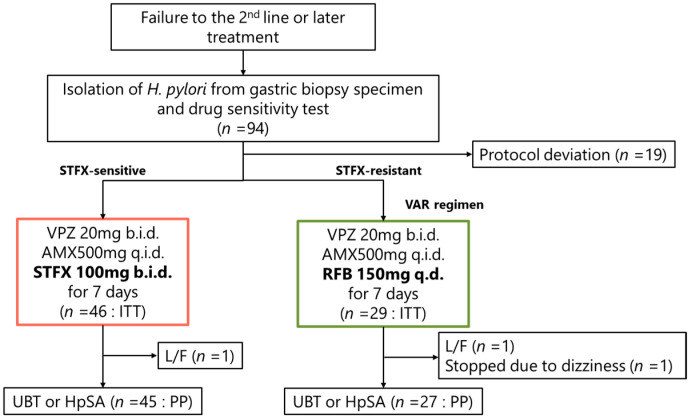
Flow diagram. ITT: intention-to-treat; PP: per-protocol; UBT: the ^13^C urea breath test; HpSA: *H. pylori* stool antigen test; L/F: lost to follow-up; VPZ: vonoprazan; AMX: amoxicillin; STFZ: sitafloxacin; RFB: rifabutin; b.i.d.: twice daily; q.i.d.: four times daily; q.d.: once daily.

Altogether, 94 patients who underwent *H. pylori* isolation and antimicrobial susceptibility testing after failure of second-line eradication therapy were screened for study eligibility (Figure 1). Among them, 19 patients were excluded due to protocol deviations, including treatment regimens inconsistent with the predefined algorithm. The remaining 75 patients received individualized therapy according to the predefined protocol. In the ITT population, one patient in the VAS group and one in the VAR group were lost to follow-up, and one patient in the VAR group discontinued therapy because of dizziness. Consequently, 72 patients were included in the PP analysis.

### 3.2. Baseline Characteristics

The baseline demographics and clinical characteristics of the study population are summarized in Table 1. The patients’ mean age was 47.7 ± 12.1 years, and 46.0% were male. Of the 75 patients, 46 (61.3%) were infected with sitafloxacin-sensitive strains and received the VAS regimen, whereas 29 (38.7%) were sitafloxacin-resistant and received the VAR regimen. All *H. pylori* isolates were sensitive to rifabutin. Amoxicillin resistance was observed in 6 (13.0%) and 14 (48.3%) patients in the VAS and VAR groups, respectively, with the VAR group showing a significantly higher resistance rate. No other demographic or clinical parameters differed significantly between the two groups.

### 3.3. Eradication Outcomes

**Figure 2:**
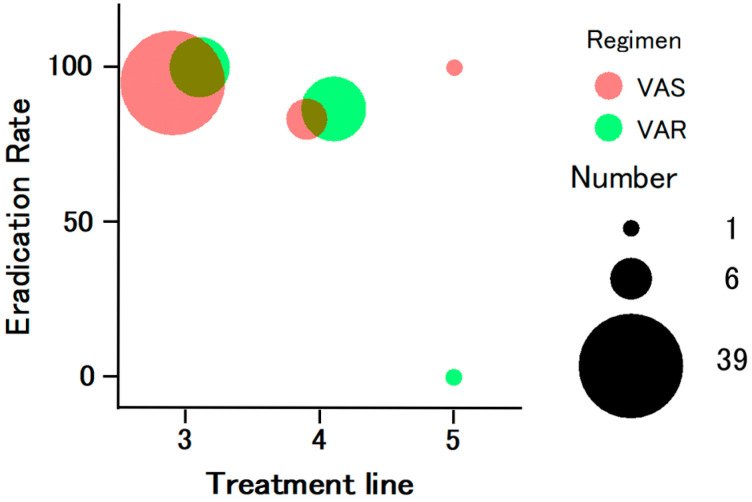
Eradication rates according to the treatment line in the VAS and VAR groups (bubble chart). Each bubble represents the eradication rate for a given treatment line, and the bubble size reflects the number of patients in each category. The eradication rates were 96.2% for third-line therapy, 85.7% for fourth-line therapy, and 50.0% for fifth-line therapy. Although the number of fifth-line cases was small and precluded a definitive evaluation, both the VAS and VAR regimens achieved favorable eradication outcomes in the third- and fourth-line therapies.

The overall eradication rates were 92.0% (69/75; 95% CI, 83.4–97.0) and 95.8% (69/72; 95% CI, 88.3–99.1) in the ITT and PP analyses, respectively. A subgroup analysis showed eradication rates of 93.5% (43/46) and 89.7% (26/29) in the VAS and VAR groups, respectively. When stratified by treatment line (Figure 2), the eradication rates were 96.2% (50/52) for third-line therapy, 85.7% (18/21) for fourth-line therapy, and 50.0% (1/2) for fifth-line therapy. When the sitafloxacin MIC was 0.06 µg/mL, the eradication rate was 71.4% (5/7), whereas all 38 cases with MIC values of ≤0.03 µg/mL achieved successful eradication in the PP analysis. No significant difference in eradication rate was observed between the VAS and VAR groups.

### 3.4. Adverse Events

Adverse events occurred in 17.3% (13/75) of all treated patients (Table 2). The most frequently reported events were mild diarrhea, abdominal discomfort, and taste disturbance. One patient (1.3%) in the VAR group discontinued therapy because of dizziness, which resolved spontaneously after cessation. No serious adverse events were observed, and no patient required hospitalization due to adverse drug reactions.

## 4. Discussion

The present multicenter retrospective study demonstrated that susceptibility-guided vonoprazan-based triple therapy achieved high eradication rates and good tolerability in patients requiring third-line or subsequent *H. pylori* eradication therapy. The overall eradication rate exceeded 90% in both the ITT and PP analyses, confirming the effectiveness of individualized therapy using VAS or VAR regimens based on the antimicrobial susceptibility results.

In Japan, empirical third-line therapies—such as vonoprazan-based triple therapy with rifabutin or sitafloxacin—have shown variable eradication rates ranging from 70% to 90% [9,22,29,30,31,32,33,34,38,48,49,50,51,52,53,54,55,56,57,58,59]. The present susceptibility-guided approach achieved eradication rates of 92.0% (ITT) and 95.6% (PP), which are equal to or superior to these empirical outcomes. This highlights the clinical value of tailoring antibiotic selection based on the results of susceptibility testing to optimize treatment efficacy and avoid unnecessary use of ineffective drugs.

Previous studies have shown that *H. pylori* strains lacking *gyrA* mutations—rather than merely those with low sitafloxacin MICs—are almost universally eradicated by the sitafloxacin triple regimens [31,52,54]. In the present study, two cases of VAS failure had a sitafloxacin MIC value of 0.06 µg/mL, which is close to the cutoff value, suggesting that these strains may have harbored undetected *gyrA* mutations [60]. Indeed, among the patients with an MIC value of 0.06 µg/mL, the eradication rate was 71.4% (5/7), whereas all 38 patients with an MIC value of ≤0.03 µg/mL achieved successful eradication. These findings imply that when stratification is based on phenotypic susceptibility, patients with an MIC value of ≤0.03 µg/mL, who can be regarded as reliably *gyrA*-negative, should preferentially receive the VAS regimen, whereas those with an MIC value of ≥0.06 µg/mL may benefit from switching to the VAR regimen. Although direct *gyrA*-based classification could theoretically provide greater precision, susceptibility-guided stratification using these MIC thresholds appears to be a practical and effective approach in clinical settings. Furthermore, with the emerging availability of gastric-fluid polymerase chain reaction testing capable of rapidly detecting *gyrA* mutations, the integration of genotypic information into clinical algorithms may soon allow even more refined individualized eradication strategies [42,61].

A potential counterargument is that, given the near-universal rifabutin susceptibility, empirical VAR treatment alone may be sufficient. However, previous studies have shown that even when *H. pylori* strains are phenotypically susceptible to rifabutin, empirical VAR regimens do not consistently achieve eradication rates exceeding 90%, suggesting that susceptibility alone does not guarantee optimal outcomes [34]. Therefore, the present susceptibility-guided algorithm—selecting VAS for *gyrA*-negative (sitafloxacin-sensitive) strains and VAR for resistant ones—provides a more reliable and rational strategy to maximize the treatment success.

From a clinical perspective, the present findings support the use of a simple susceptibility-guided algorithm for treatment selection in later-line settings. By directing patients with sitafloxacin-susceptible strains to the VAS regimen and those with resistant strains to the VAR regimen, this approach provides a pragmatic framework for individualized therapy that aligns with principles of antibiotic stewardship. Importantly, this strategy is readily applicable in real-world practice, as it relies on phenotypic susceptibility testing that is already available in many clinical settings. The multicenter design of the present study further enhances the generalizability of these findings beyond a single specialized institution.

Despite the favorable outcomes observed, several issues warrant further investigation. Integrating genotypic resistance testing, such as rapid detection of *gyrA* mutations using gastric fluid- or biopsy-based PCR assays, may further refine treatment stratification [62,63,64]. Prospective multicenter studies are also needed to validate the proposed algorithm in larger and more diverse populations and to compare susceptibility-guided strategies directly with empirical rescue therapy. In addition, evaluating cost-effectiveness and patient-reported outcomes will be important, particularly in healthcare systems where third-line eradication therapy is not uniformly reimbursed.

Finally, several limitations should be acknowledged. The retrospective design and relatively limited sample size may restrict the generalizability of the findings, and the absence of a direct comparison with empirical therapy precludes definitive conclusions regarding superiority. Moreover, the higher prevalence of amoxicillin resistance in the VAR group may have influenced eradication outcomes. Future prospective studies incorporating both phenotypic and genotypic resistance assessments are warranted to establish a standardized framework for individualized eradication therapy in refractory *H. pylori* infection.

## 5. Conclusions

Susceptibility-guided vonoprazan-based triple therapy achieved high eradication rates and a good safety profile in patients requiring third-line or later *H. pylori* eradication therapy. Stratification based on the sitafloxacin MIC values—treating patients with an MIC value of ≤0.03 µg/mL with the VAS regimen and those with an MIC value of ≥0.06 µg/mL with the VAR regimen—may further optimize individualized therapy. Future prospective studies incorporating both phenotypic and genotypic (*gyrA*) assessments are warranted to refine this strategy and establish an evidence-based framework for personalized *H. pylori* eradication therapies.

## Figures and Tables

**Table 1:** Participants’ demographics.

	Total (*n* = 75)	7-Day VAS (*n* = 46)	7-Day VAR (*n* = 29)	*p*-Value
Age (years, mean ± SD)	47.7 ± 12.1	46.6 ± 11.8	51.9 ± 9.7	0.720 ^†^
Sex (male/female)	23/27	20/26	13/16	1.000 ^‡^
Resistance to STFX (MIC: ≥0.12 μg/mL), *n* (%)	29 (38.7)	0	29 (100)	0.000 ^‡^
Resistance to RFB (MIC: ≥0.25 μg/mL), *n* (%)	0	0	0	N/A ^‡^
Resistance to AMX (MIC: ≥0.06 μg/mL), *n* (%)	20 (26.7)	6 (13.0)	14 (48.3)	0.001 ^‡^
Resistance to CLR (MIC: ≥1 μg/mL), *n* (%)	69 (92.0)	42 (91.3)	27 (93.1)	1.000 ^‡^
Resistance to MTZ (MIC: ≥8 μg/mL), *n* (%)	60 (80.0)	36 (78.3)	24 (82.8)	0.770 ^‡^

All patients with STFX-sensitive strains were treated with the VAS regimen, whereas those with STFX-resistant strains received the VAR regimen. The prevalence of AMX resistance was significantly higher in the VAR group, whereas no other significant differences were observed between the two groups. AMX: amoxicillin; STFX: sitafloxacin; RFB: rifabutin; CLR: clarithromycin; MTZ: metronidazole. † Student’s *t*-test; ‡ Fisher’s exact test.

**Table 2:** Adverse events during individualized *H. pylori* eradication therapy.

	Total	7-Day VAS	7-Day VAR	*p*-Value
ITT population, *n*	74	45	29	
Total adverse events, *n* (%)	13 (17.6)	6 (13.3)	7 (24.1)	0.348 ^‡^
Treatment discontinuation, *n* (%)	1 (1.4)	0	1 (8.3)	0.392 ^‡^
Soft stool, *n* (%)	3 (4.1)	3 (6.7)	0	0.275 ^‡^
Diarrhea, *n* (%)	2 (2.7)	2 (4.4)	0	0.517 ^‡^
Fatigue, *n* (%)	1 (1.4)	0	1 (8.3)	0.392 ^‡^
Headache, *n* (%)	2 (2.7)	1 (2.2)	1 (8.3)	1.000 ^‡^
Nausea, *n* (%)	1 (1.4)	0	1 (3.5)	0.392 ^‡^
Fever, *n* (%)	1 (1.4)	0	1 (3.5)	0.392 ^‡^
Conjunctival injection, *n* (%)	1 (1.4)	0	1 (3.5)	0.392 ^‡^
Skin rash, *n* (%)	1 (1.4)	0	1 (3.5)	0.392 ^‡^

Adverse events occurred in 17.6% of patients, with no significant differences between the VAS and VAR groups. One patient was excluded from adverse event analysis due to missing safety data. ITT: intention-to-treat, ‡ Fisher’s exact test.

## Data Availability

The data presented in this study are available on request from the corresponding author. The data are not publicly available due to privacy and ethical restrictions.
